# Development and Characterization of Novel Porous 3D Alginate-Cockle Shell Powder Nanobiocomposite Bone Scaffold

**DOI:** 10.1155/2014/146723

**Published:** 2014-07-07

**Authors:** B. Hemabarathy Bharatham, Md. Zuki Abu Bakar, Enoch Kumar Perimal, Loqman Mohamed Yusof, Muhajir Hamid

**Affiliations:** ^1^Biomedical Sciences Programme, School of Diagnostic and Applied Sciences, Faculty of Health Sciences, Universiti Kebangsaan Malaysia, Jalan Raja Muda Abdul Aziz, 50300 Kuala Lumpur, Malaysia; ^2^Department of Veterinary Preclinical Sciences, Faculty of Veterinary Medicine, Universiti Putra Malaysia (UPM), 43400 Serdang, Selangor Darul Ehsan, Malaysia; ^3^Institute of Biosciences, Universiti Putra Malaysia (UPM), 43400 Serdang, Selangor Darul Ehsan, Malaysia; ^4^Department of Biomedical Sciences, Faculty of Medicine and Health Sciences, Universiti Putra Malaysia, 43300 Serdang, Selangor, Malaysia; ^5^Department of Veterinary Clinical Studies, Faculty of Veterinary Medicine, Universiti Putra Malaysia (UPM), 43400 Serdang, Selangor Darul Ehsan, Malaysia; ^6^Department of Microbiology, Faculty of Biotechnology and Biomolecular Sciences, Universiti Putra Malaysia (UPM), 43400 Serdang, Selangor Darul Ehsan, Malaysia

## Abstract

A novel porous three-dimensional bone scaffold was developed using a natural polymer (alginate/Alg) in combination with a naturally obtained biomineral (nano cockle shell powder/nCP) through lyophilization techniques. The scaffold was developed in varying composition mixture of Alg-nCP and characterized using various evaluation techniques as well as preliminary *in vitro* studies on MG63 human osteoblast cells. Morphological observations using SEM revealed variations in structures with the use of different Alg-nCP composition ratios. All the developed scaffolds showed a porous structure with pore sizes ideal for facilitating new bone growth; however, not all combination mixtures showed subsequent favorable characteristics to be used for biological applications. Scaffolds produced using the combination mixture of 40% Alg and 60% nCP produced significantly promising results in terms of mechanical strength, degradation rate, and increased cell proliferation rates making it potentially the optimum composition mixture of Alg-nCP with future application prospects.

## 1. Introduction

The unique physical, mechanical, and chemical characteristics of polymers make them interesting candidates for scaffold fabrication. Found either as natural or synthetic polymers as well as degradable or nondegradable, this interesting group of materials forms a category of substitutes that differ from others [1] and by far the widest group of existing graft substitute materials. A scaffold fabricated for the intentions of being used as a bone substitute material should be produced from a highly biocompatible material with adequate physical and mechanical properties without eliciting an immunological or clinically detectable foreign body reaction [2]. Ideally, the scaffold should also provide sufficient structural integrity, high surface area for cell-material interaction while degrading in a rate proportional to the regeneration of new bone [3]. The scaffold basically sets the stage as an extracellular matrix that provides a three-dimensional architecture capable of performing significant function.

The abundant availability and relatively low cost of natural polymers makes it an attractive option for the fabrication of bone scaffolds [4]. One such naturally occurring polysaccharide that is widely studied in tissue engineering and drug delivery system is alginate. The cross-linking and gelation of the alginate can be easily tailored to produce the desired characteristics such as porosity and mechanical stability. Typically, the presence of a divalent cation such as calcium ions is sufficient to produce the cross-linking action involving the alginate monomers. This action often results in the egg-box model that displays sufficient porosity and pore size ideal as a bone tissue scaffolding material.

Recent researches on polymer based grafts are focusing on the formation of composite based grafts in order to help improve the mechanical behavior of the polymers. Some of the recent studies that were carried out on polymer based grafts includes poly (D,L-lactide)/nanohydroxyapatite composite [5], fibrin and poly(lactic-co-glycolic acid) hybrid scaffold [6], alginate/nanoTiO_2_ needle composite scaffolds [7], hydroxyapatite/chitosan-alginate composites [8], and others. Though results from these studies are encouraging, various concerns on sufficient mechanical stability, degradability of the materials, and its subsequent inflammatory response of the native tissues could be highlighted. These drawbacks provide avenues for improvement through better material combinations during biocomposite scaffold fabrications.

The cockle belonging to the species of* Anadara granosa* is a type of sea mollusks widely consumed in South East Asia. The shells represent a large portion of waste products after the mussels are consumed. Studies by Zakaria et al. [9] and others [10, 11] have shown the potential use of the cockle shell based calcium carbonate powder as a source of biomineral for bone tissue applications. The powder obtained from the shells nacreous materials are shown to possess high similarities with coral exoskeletons [10]. Similar to corals, cockle shells are also found to consist purely of the aragonite form of calcium carbonate polymorph, which is denser in nature giving it an added advantage to be incorporated, resolved, and then replaced by bones over time compared to the other forms of calcium carbonate polymorphs [12]. 

The current trends in bone scaffold fabrication highlight the use of calcium phosphate based materials with very few studies done using calcium carbonates. The calcium phosphate ceramic based bone graft substitutes form one of the largest groups of commercially available grafting materials that include some common compositions of calcium hydroxyapatite (HAp), *β*-tricalcium phosphate (*β*-TCP), silicate calcium phosphate (Si-CaP), and bioactive glasses [13]. Unlike the naturally occurring hydroxyapatite in the bone, the commercially produced HAp material from calcium phosphates often possesses limitations such as low solubility and slow* in vivo* resorbability [14]. Although calcium phosphate based materials provide excellent osteoconductiveness, the long term presence of the material within the biological system was found to limit the formation of the native bones [15]. Emerging studies on calcium carbonate based grafting material on the other hand may be manipulated in order to address the limitations of calcium phosphate based bone grafts in the near future.

With the potential use of alginate as a scaffold being documented as an effective surgical construct for prevascularized bone grafting [16] or used in combination with other materials such as chitosan [17] and hydroxyapatite [18] to produce potentially new bone scaffold materials, we attempted for the first time to use this polymeric material in combination with cockle shell powder to develop and characterize a novel three-dimensional calcium carbonate based nanobiocomposite scaffold with potential bone grafting properties. The scaffold is constructed in varying compositions and tested in a series of short-term studies in order to determine the most ideal composition ratio for further application purposes.

## 2. Materials and Methods

### 2.1. Alginate Solution Preparation

Sodium alginic acids were purchased from Sigma UK (Cat number 180947 MW 102000–209000, M : G ratio 1.56). Alginate hydrocolloid solutions consisting of 20%, 40%, 60%, 80%, and 100% (w/v) were prepared by gradually dissolving preweighed powder in 10 mL of deionized water under constant stirring at 600 rpm till completely dissolved.

### 2.2. Nano Cockle Shell Powder Preparation

Cockle shell powder was prepared according to the methods of Zakaria et al. [9]. The micron sized powder obtained was then converted to nanoparticles using a biomineralization catalyst (dodecyl dimethyl betaine) through a simple chemical method involving mechanical stirring of the cockle shell powder in the presence of the catalyst at room temperature. The resulting slurry is filtered and oven dried overnight at 100°C prior to the characterization studies. The method produced cockle shell nanoparticles with an average size of 30 ± 5 nm determined through scanning electron and transmission electron microscopic examination. The detail procedure of the nano cockle shell synthesized and used for this study is given by Islam et al. [11].

### 2.3. Scaffold Development

Alginate nano cockle shell powder scaffolds (Alg-nCP) were developed by mixing preweighed nano powder into prepared alginate hydrocolloid solution under constant stirring on a homogenize stirrer machine at 600 rpm until a homogenized smooth slurry is obtained. The scaffold mixture was prepared in five different compositions of Alg : nCP; 80 : 20, 60 : 40, 40 : 60, 20 : 80, and 100 : 0 (w/v) that are designated as scaffolds 1, 2, 3, 4, and 5, respectively. The completely homogenized mixture was poured into a custom made cylindrical mold (4 cm height ×1 cm diameter), labeled, allowed to set, and freezed at −20°C for 24 hours. The scaffolds were then transferred to a freeze dryer machine for lyophilization till completely dried (24 hours at −50°C). The lyophilized scaffolds were then removed from the molds and cross-linked by soaking in 1% CaCl_2_ solution for 20 minutes. This was followed by three times washing with deionized water and an overnight soaking in deionized water to remove any unbound CaCl_2_. The scaffolds were then lyophilized in a freeze dryer machine for 24 hours and stored in a sterile container prior to use.

### 2.4. Scanning Electron Microscopy (SEM)

Microstructure of the prepared scaffolds was observed by scanning electron microscopy (VPSEM-JEOL 1455, Germany). The scaffolds were cut into smaller sections, fixed on stubs at various angles, and sputter coated with gold prior to observation. Average pore diameter of each sample was measured based on 30 measurements taken at various sites on the micrograph picture from three replicates per scaffold.

### 2.5. Porosity Evaluation

Porosity of the prepared scaffolds was determined through liquid displacement method according to the methods of Y. Zhang and M. Zhang [19] using ethanol. The scaffolds were first cut into smaller circular discs measuring 1 cm in diameter and 1 cm thickness. Initial weight (*W*_0_) and the volume (*V*) of six samples per scaffold were measured prior to immersion. The samples were immersed in ethanol for 48 hours to allow complete saturation after which it is weighed and noted as *W*_1_. The porosity of the scaffold was calculated according to the following formula with *ρ* representing the density of ethanol:

(1) $$\begin{array}{c} \text{porosity }\left(\%\right)=\frac{\left(W_{1}-W_{0}\right)}{\rho V}\times 100\%. \end{array}$$

### 2.6. Swelling Evaluation

The swelling study was carried out according to the methods of Soumya et al. [20] in order to understand the absorption and diffusion of medium and nutrients into the scaffold which is essential for cell viability. The swelling behavior of the scaffolds was studied by determining the percentage of medium uptake by each scaffold. Previously weighed scaffolds measuring 1 cm diameter and 1 cm thickness were immersed in PBS solution pH 7.4 in a preweighed container. At a given interval the solutions were carefully withdrawn and the wet weights of the samples were measured. The percentage of medium uptake was calculated using the following formula for six replicates from each scaffold:

(2) percentage  of  medium  uptake =final  weight−initial weightinitial weight×100%.

### 2.7. Degradation Evaluation

Degradation of the composite scaffolds was studied according to the methods of Peter et al. [21]. Six samples from each scaffold measuring 1 cm in diameter and 1 cm thickness were incubated in PBS solution pH 7.4 containing 10 000 U/mL lysozyme (Sigma 62970) at 37°C for 14 days. The solution was changed every three days to ensure continuous enzyme activity. Initial weights of the scaffolds are recorded as *W*_0_ and after 14 days the scaffolds were carefully removed and freeze dried till completely dried (24 hours at −50°C). The scaffolds were then weighed and the dry weight was noted as *W*_1_. The degradation rate was then calculated according to the following formula:

(3) $$\begin{array}{c} \text{degradation}\;\left(\%\right)=\frac{W_{0}-W_{1}}{W_{0}}\times 100\%. \end{array}$$

### 2.8. Mechanical Testing

Compressive mechanical strength and modulus of scaffolds were tested using an Instron 4505 mechanical tester with 10 kN load cells [17]. Six samples from each scaffold were prepared in circular discs of 1.0 cm in diameter and 1.2 cm thickness. A crosshead speed of 0.5 mm/min was used and the load was applied until the samples were compressed to approximately 100% of its original height.

### 2.9. Fourier Transform Infrared (FTIR) Analysis

The chemical functionality of the scaffold composites was determined by the spectroscopic method using a Fourier transform infrared (FTIR) spectrophotometer (Perkin Elmer) over the range of 400 cm^−1^ to 4000 cm^−1^ using 1-2 g of samples prepared through UATR methods.

### 2.10. X-Ray Diffraction Analysis

The wide-angle X-ray diffraction was performed at room temperature to characterize the nature of amorphous and crystalline. All crystalline phases present were identified using diffractometer with the diffraction angles from 0 to 70°C. The analysis was performed in 2 g of powdered scaffolds.

### 2.11. *In Vitro* Culture and Scaffold Extract Preparation

MG63 osteoblast-like cells obtained from American Type Culture Collection (ATCC number CRL-1427) were grown in the cell culture facility under a controlled atmosphere according to the manufacturer's protocol. The cells from a single vial were seeded into a T25 culture flask containing Dulbecco's modified Eagle medium (DMEM: PAA E15-810) supplemented with 10% fetal bovine serum (FBS: Sigma F4135) and 1% penicillin/streptomycin (PAA P11-010) at 37°C temperature and 5% CO_2_. The medium was changed every 2-3 days and cells were routinely split 1 : 2 every 3-4 days at 80% confluence. The cells were detached using trypsin/EDTA (0.25% w/v trypsin/0.02% EDTA, Sigma), concentrated by centrifugation at 800 rpm for 5 minutes, and resuspended in fresh medium. Extracts from developed scaffolds were prepared according to the methods of Peter et al. [21] for the purposes of MTT assay and live/dead cell staining. Scaffolds sterilized through autoclaving and UV irradiation for 20 minutes were incubated in 20 mL of DMEM culture media in separate flasks at 37°C for 24 hours in order to obtain the scaffold extracts. The leachable from each scaffold was then collected into individually labeled sterile falcon tubes and stored at 4°C for further use.

### 2.12. MTT (3-Dimethylthiazo-2,5-diphynyltetrazolium Bromide) Colorimetric Assay

MTT assay was carried out according to the methods of Peter et al. [21]. The MG63 osteoblast cells were seeded on a 96-well plate at a density of 1 × 10^4^ cells/well. The cells were incubated under standard culturing conditions for 24 hours to allow initial attachments. After 24 hours, the medium was removed from each well and replaced with medium containing the scaffold extracts and incubated further for 48 hours. Following the incubation period, the medium with scaffold extracts was removed from each well and replaced with fresh medium containing 10% of MTT solution. The cells were then incubated at 37°C for additional 4 hours in order to perform the standard MTT assay. After 4 hours the medium was removed and the reaction was terminated with the addition of 100 *μ*L dimethyl sulfoxide (DMSO) as a solubilization buffer to each well to dissolve the formazan crystals. The lysate absorbance was then read at a wavelength of 495 nm using a microplate reader and the results were expressed as absorbance reading from each well. The assay was conducted in duplicates with eight replicates per scaffold. One row from each plate was designated for control and was cultured with normal culture medium.

### 2.13. Live/Dead Cell Staining

MG63 osteoblast cells at a density of 1 × 10^5^ cells/well were cultured in a 6-well plate. The cells were cultured under standard culturing conditions for 24 hours to allow initial attachments. The medium from each well was removed after 24 hours and replaced with medium containing the scaffold extracts. The cells were then allowed to grow in the scaffold extracts for 5 days with two time's medium change. After 5 days, the cells were trypsinized and detached in normal medium containing serum. The detached cells were collected in a sterile falcon tube and spun at 800 rpm for 5 min. The resulting supernatant is then discarded leaving the cell pellets for further use. The staining solution is then prepared by adding 100 *μ*L of 1 mg/mL propidium iodide (PI, Sigma, UK) and 100 *μ*L of 1 mg/mL acridine orange (AO, Sigma, UK) to 10 mL phosphate buffer solution. The cell suspensions are then mixed 1 : 1 with the staining solution in an eppendorf tube; 10 *μ*L from the mixed suspension is then transferred unto a glass slide, cover slipped, and viewed under the fluorescence microscope (Nikon). Cells were then visually observed for live/viable cells stained green and dead/apoptotic cells stained red. The percentage of fluorescence cells were quantitated using Adobe Photoshop CS3.

### 2.14. Statistical Analysis

All quantitative results were analyzed using one-way analysis of variance (ANOVA). Results were expressed as mean ± standard deviation (SD). Post hoc tests were done for significant values (*P* < 0.05) using Tukey's multiple comparison test.

## 3. Results and Discussion

### 3.1. Scaffold Development

**Figure 1:**
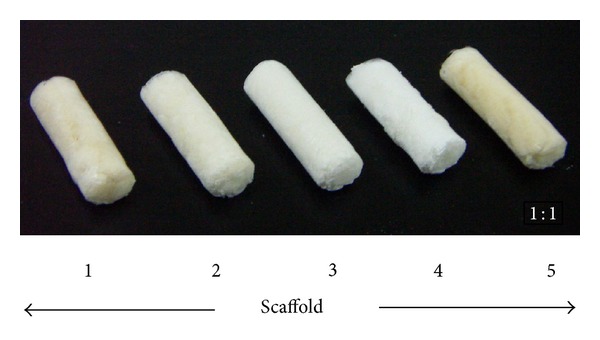
Scaffolds 1 to 5 developed in varying compositions of Alg-nCP. ∗Alg : nCP. scaffold 1 (80 :  20); scaffold 2 (60 : 40); scaffold 3 (40 : 60); scaffold 4 (20 : 80); scaffold 5 (100 : 0).

The scaffolds produced through lyophilization method and cross-linking steps using alginate produced a scaffold structure that was found to be ideal for bone tissue grafting purposes. In addition to the egg box structure produced with the cross-linking of alginate monomers, the choice of this fabrication technique produced scaffolds with excellent porosity that is contributed by the formation of ice crystals during freezing and its subsequent removal during lyophilization. The scaffolds produced were found to be rigid structures that had a sponge-like appearance. The shade of the scaffolds varied based on the content of the cockle powder, with higher content of cockle powder producing whiter scaffolds (Figure 1).

### 3.2. Scanning Electron Microscopy (SEM)

**Figure 2:**
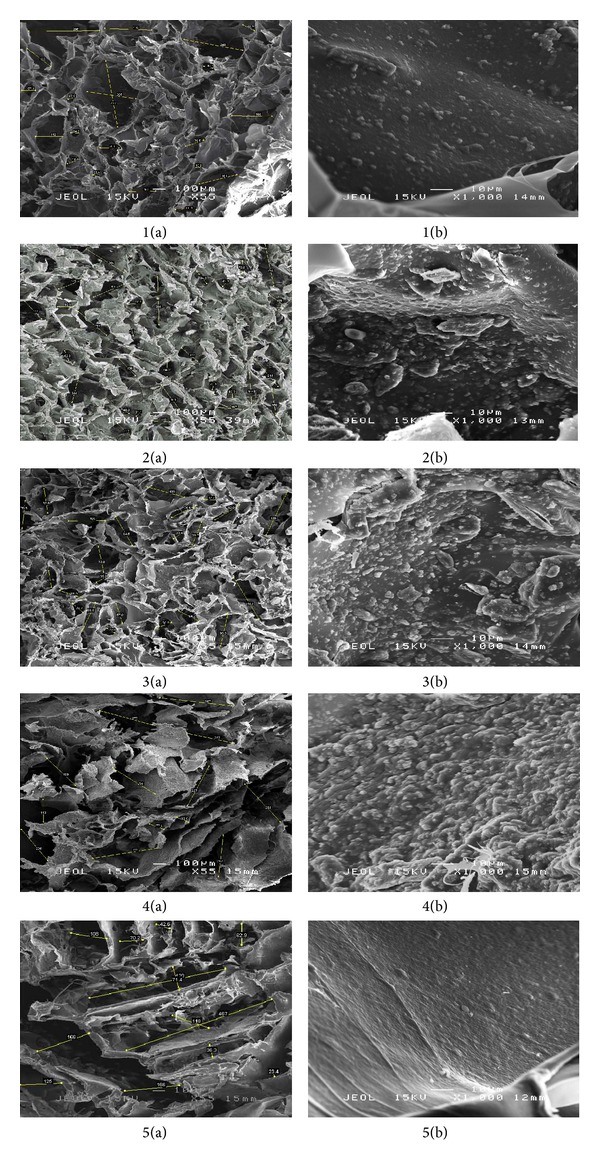
SEM images showing the morphology of Alg-nCP pore structures (a: ×55) and nano cockle shell deposition on the polymer matrix (b: ×1000) of scaffolds 1 to 5.

The results from SEM revealed the micro architecture of the scaffolds in terms of their pore sizes, diameters, interconnectivity, and arrangements within the scaffolds. The developed scaffolds regardless of their composition showed adequate pore sizes ranging from 50 to 336 *μ*m (Table 1). An ideal bone scaffold that is favorable for facilitating new bone regeneration should possess a porous structure with pore sizes ranging from 100 to 300 *μ*m [22]. This ideal pore range occurred predominantly in scaffolds 1, 2, and 3 as compared to scaffold 4 that showed visible differences in pore morphology. The high concentration of nano cockle powder in scaffold 4, however, resulted in the collapse of the alginate network structure. The typical egg box model with well-organized pore structures was not observed with the use of this composition. The presence of calcium ions contributed by the cockle powder is an important factor that forms the cross-linking of alginate molecules in order to produce the spherical porous structured network as compared to the lamellar sheet like arrangement of a pure alginate (scaffold 5).  Figure 2 shows the morphological appearance of the pore structures of the scaffolds and the nano cockle shell depositions on the alginate matrices.

### 3.3. Porosity Measurements

**Figure 3:**
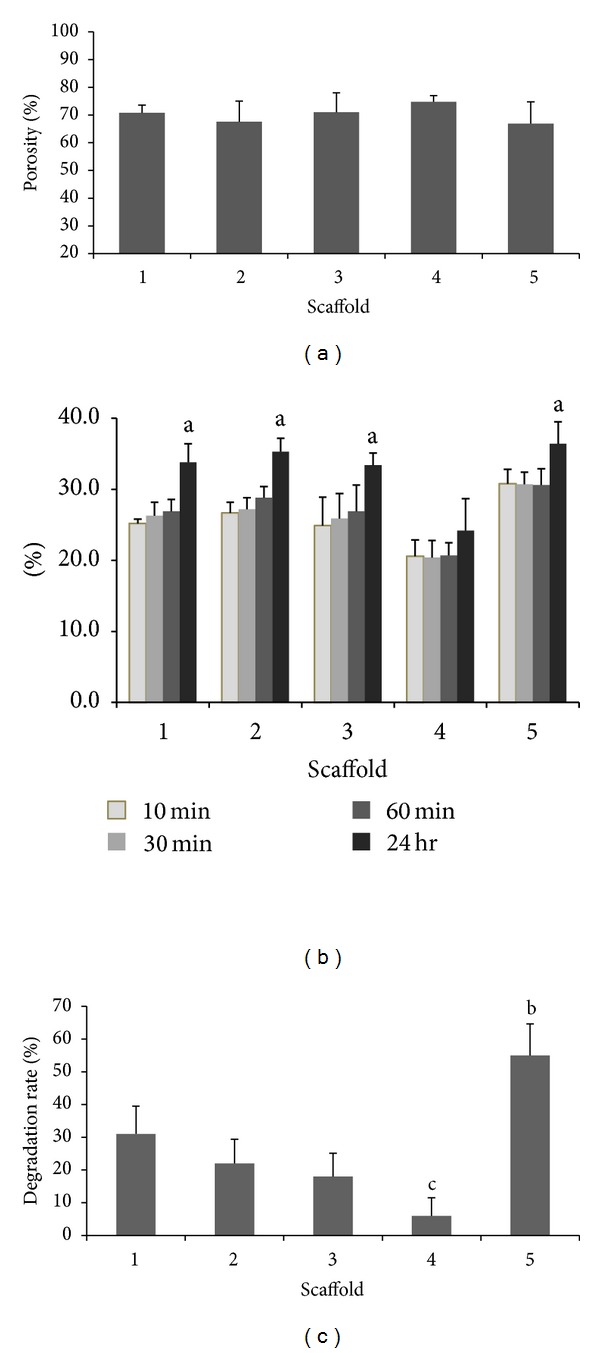
(a) Porosity study of the scaffolds. (b) Swelling studies of scaffolds; ^a^significant differences *P* < 0.05, *n* = 6. (c) Enzymatic degradation studies; ^b^significantly higher than scaffolds 1, 2, 3, and 4 at *P* < 0.05, *n* = 6, ^c^significantly lower than scaffolds 1, 2, and 5 at *P* < 0.05, *n* = 6.

A highly porous structure is known to facilitate better healing quality as it promotes cell migrations and vascularization producing a more conducive environment for new bone growth. Porosity is an important characteristic of a scaffold due to its direct linkage to the degradability, swelling aspects, and the mechanical properties of the scaffolds. The porosity of the scaffolds was found to be above 60% and was noted to increase proportionally with the nano cockle shell powder content in the composition (Figure 3(a)). Similar findings are also reported by Soumya et al. [20] that observed an increase in porosity with the addition of extracts. This phenomenon is likely due to the evaporation of water molecules (during lyophilization) that covers the aggregated nano powder particles that gets dispersed within the alginate matrix during freezing thus forming a porous structure. The higher content of nano cockle powder results in a higher amount of aggregates dispersed within the alginate matrices consequently correlating to the higher value of porosity observed in scaffold 4. Another possible explanation for the higher porosity value in scaffold 4 may be attributed to the structural collapse which has resulted in wider pore structures.

### 3.4. Swelling Evaluation

The swelling behavior of a scaffold is an important aspect that causes an increase in pore sizes that determines its practical use in facilitating the attachment and growth of cells and the subsequent new tissue regeneration [23]. In regard to swelling ratio of the scaffolds, an average of 30% of medium uptake ability within the time of study was displayed by scaffolds 1, 2, and 3 (Figure 3(b)). Despite having larger pore sizes, scaffold 4 showed the lowest medium uptake ability compared to scaffold 5 which showed otherwise. One possible explanation for this decrease could be attributed to the interaction of the nano cockle powder with the alginate network and its subsequent bonding properties. A decrease in swelling ratio is often evident when a scaffold is produced as a composite form [21]. An interesting observation from this study was that the swelling rate was found to be proportionally increased with the amount of alginates used in the composition mixture while the porosity percentage was found to be proportionally increased with the content of the nano cockle shell powder.

### 3.5. Degradation Evaluation

An important aspect in the field of tissue engineering is often attributed to the degradation behavior of a polymer-based scaffold. An ideal rate would be that which corresponds to the regeneration of new bony structure in order to provide a smooth transition [24]. A direct relationship was observed between the swelling behavior and degradation rate with scaffold 5 showing the highest degradation rate and scaffold 4 the lowest. Scaffolds 1, 2, and 3 produced intermediate results with the rate of scaffold 3 being more desirable for further applications (Figure 3(c)). The enzymatic degradation study was performed as a short term observation based upon the nature of lysozyme enzymes to break down the glycosidic linkage of the alginate network. In observing the enzymatic degradation study, it could be justified that the breakage of linkage within the alginate network occurs at an accelerated rate with the increase in fluid uptake thus causing the loss of network structure within a few days of soaking. The low concentration of alginate in scaffold 4 results in its structural integrity and stability. A desirable characteristic of a scaffold would be a degradation rate that is neither too fast nor too slow for it to be rendered workable.

### 3.6. Mechanical Testing

**Figure 4:**
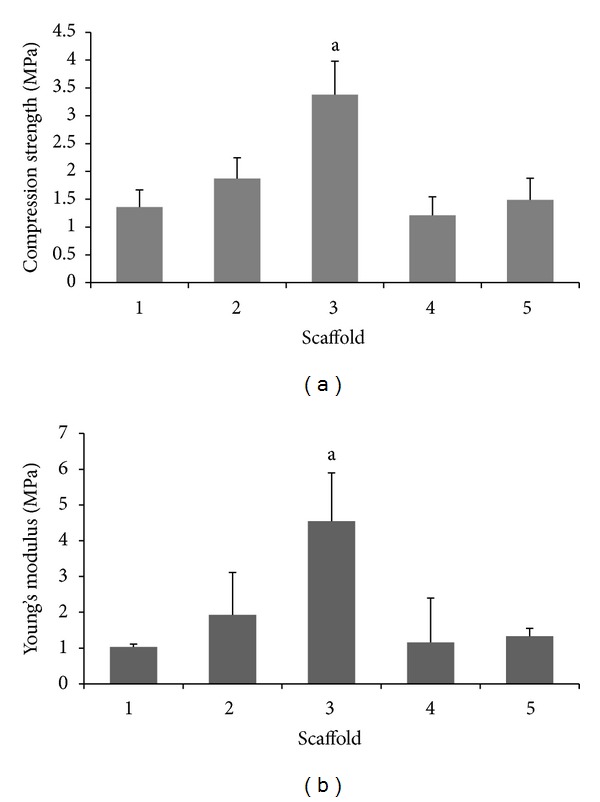
Compressive strength and Young's modulus of scaffolds tested under a 10 kN load. ^a^significantly higher than scaffolds 1, 2, 4, and 5 at *P* < 0.05, *n* = 6.

An implanted scaffold should display certain level of mechanical strength in order to act as a temporary support material. Scaffolds designed for bone tissue engineering should possess the ability to withstand physical stress in order to facilitate the tissue regeneration process. Physical observations on the scaffolds showed a lack of brittleness in all samples tested. However plastic deformity with no signs of physical fractures or breaking at the tested load was observed in all scaffolds except scaffold 4 that was found to have lost its intact structure. The stress-strain relations were carried out from which the yield strength and Young's modulus were evaluated (Figure 4). The results from this study indicated an increase in both the mechanical properties and Young's modulus of the scaffolds that were incorporated with the nano cockle powder compared to pure alginate scaffolds (scaffold 5). The increased trend of mechanical properties was found to be proportional with the increase in the nano cockle powder content in the scaffold's composition. An exceptional was observed with scaffold 4 that showed a reduction in its mechanical strength and modulus despite having the highest content of nano cockle powder. This is likely to be attributed to the high porosity of scaffold 4 that has eventually caused a tradeoff in its mechanical properties. The elongated pore shapes of scaffold 4 as observed through SEM micrographs could be another contributing factor to the weak scaffold structure as spherical pores has better tendency to resist larger compression loads [25]. In this study, scaffold 3 showed a favorable mechanical property that was found to range between the spongy bone structure of 2 and 12 MPa [26] with a compressive strength of 3.4 MPa, making this composition most suitable in terms of mechanical properties.

### 3.7. Fourier Transform Infrared (FTIR) Analysis

**Figure 5:**
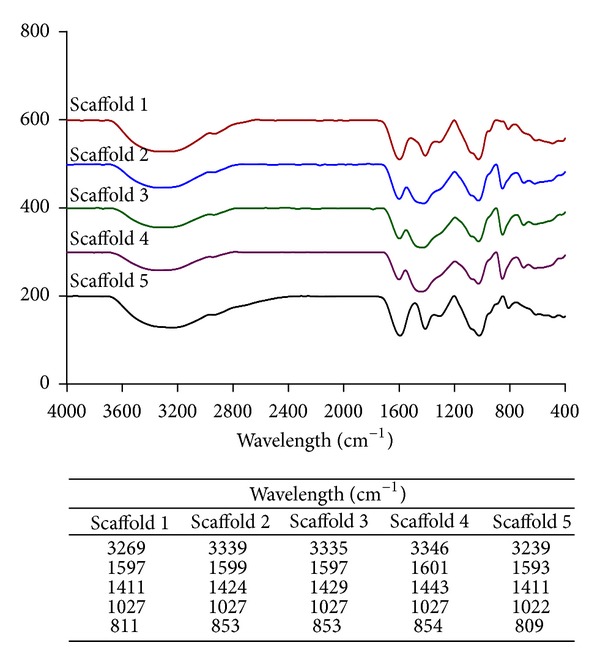
FTIR spectra and summary of dominant peaks of scaffolds.

Studying the chemical interaction of the materials in the scaffold composite is an important aspect that could help in the modification and enhancement of the scaffold properties. Studies such as FTIR and XRD are useful tools in revealing the physiochemical properties of the materials and the subsequent chemical interactions. In observing the FTIR spectra of the scaffolds (Figure 5), it was obvious that scaffolds 1 to 4 presented with a similar pattern of spectra due to the presence of the same materials in varying composition. The differences could be attributed to the sharpness and broadness of the peaks produced. The stretching vibration of carbonyl (C=O) bonds of alginate produces strong peaks in the region of 1680–1597 cm^−1^. The stretching mode of hydroxyl groups (–OH) and absorbed water molecules are indicated by the presence of absorption bands in the 3250–3400 cm^−1^ region [27, 28]. The characteristic peak of the –OH group of alginate observed in scaffold 5 at 3239 cm^−1^ was observed to have gradually shifted in scaffolds 1, 2, 3, and 4. A gradual shift of carboxyl (–COOH) bands of alginate was also noted from 1411 cm^−1^ as observed in the spectra of scaffold 5 to 1424, 1429, and 1443 cm^−1^ in scaffolds 2, 3, and 4, respectively. These shifts were found to be proportional to the increasing amount of nano cockle shell powder in the scaffolds and indicates the formation of the ionic interaction between the positively charged calcium and the negatively charged carboxyl group of alginate [20, 29]. It is this ionic interaction that acts as a contributing factor to many of the characteristics of the scaffolds, including the mechanical properties and degradation behaviors. A common band at the region of 1027 cm^−1^ was observed in the spectra of all scaffolds indicating the stretching of the C–O groups of alginate. Bands occurring at the region of 853 cm^−1^ (scaffolds 2 and 3) and 854 cm^−1^ (scaffold 4) indicates the CO_3_^−2^ groups of aragonite. These peaks observed correspond to an important aspect on the characteristics of the calcium carbonate present in the developed scaffolds.

### 3.8. X-Ray Diffraction Analysis

**Figure 6:**
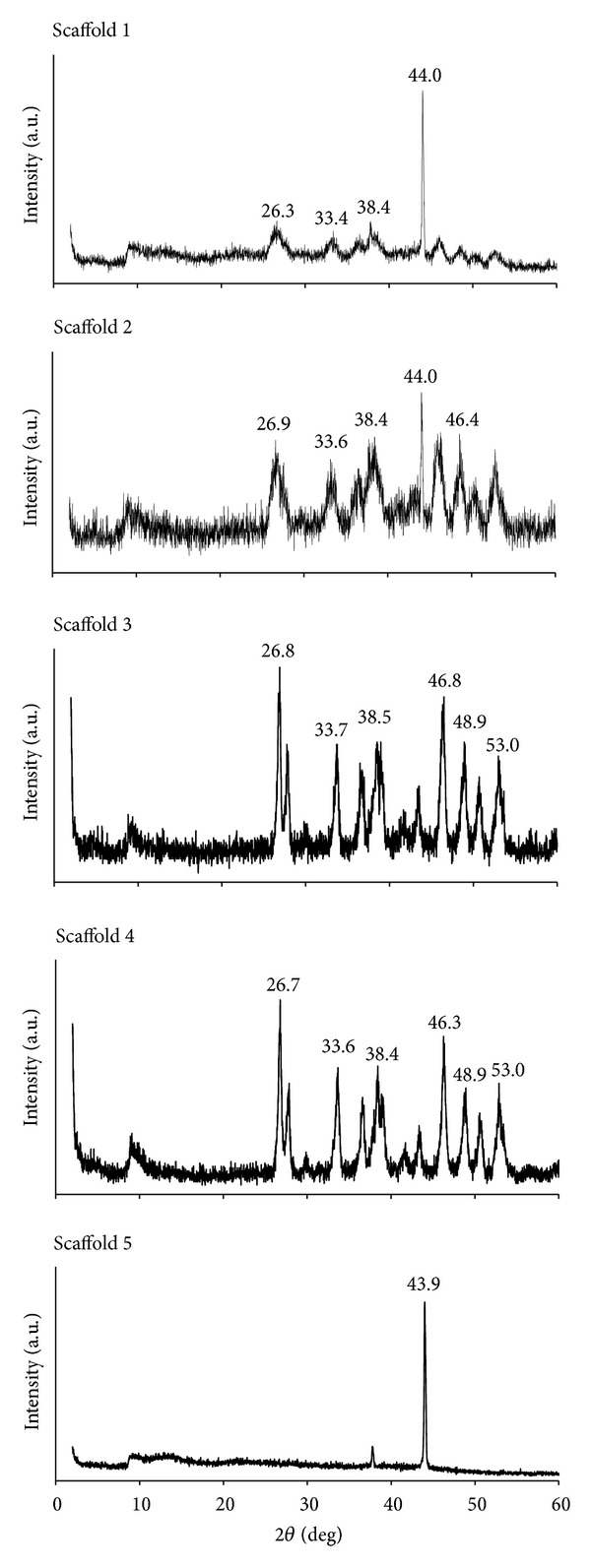
XRD spectrum of scaffolds.

The XRD analysis performed reveals important information about the mineral phase of the materials present in the scaffolds. The information obtained from the spectrums (Figure 6) confirmed the presence of the characteristic peaks of aragonite. The peaks observed over the range of 26.3 to 26.9, 33.4 to 33.6, and 46.4, 46.3, and 46.8 of scaffolds 2, 3, and 4, respectively, correspond to the characteristic peaks of the aragonite form of calcium carbonate (Joint Committee of Powder Diffraction Society (JCPDS) file number 00-001-0628 (figure not shown)). These peaks were not present in the spectrum of scaffold 5, which showed a single dominant peak of calcium cross-linked sodium alginate occurring at 43.9. Given the higher content of alginate in scaffolds 1 and 2, this dominant peak was also observed to occur at 44.0 in its spectrum but the intensity was not obvious in the spectrum of scaffolds 3 and 4, which showed additional dominant peaks of calcium carbonate at 48.9 and 53.0. Apparently, all scaffolds containing nano cockle shell powder appeared to be presented in the aragonite form of calcium carbonate, which was displayed as the only mineral phase to be present with no impurities or additional material produced during the development process.

### 3.9. MTT (3-Dimethylthiazo-2,5-diphynyltetrazolium Bromide) Colorimetric Assay

**Figure 7:**
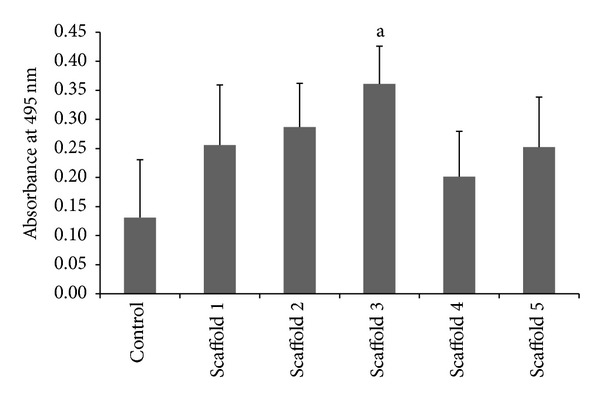
MTT calorimetric assay. ^a^significantly higher than scaffolds 1, 2, 4, and 5 and control at *P* < 0.05.

The MTT colorimetric assay is often used as a first-line test for biocompatibility with a dual purpose of quantifying the cytotoxic effect of the scaffold materials towards the cells as well as an indicator for the proliferation rate of the cells [30]. The ability of the cells to proliferate and grow in the scaffold extracts acts as a direct indicator of the absences of cytotoxic effect from the products leaching out from the scaffold materials. Results from the study (Figure 7) showed an increasing trend in cell proliferation rate with the increase in the content of nano cockle shell powder in the scaffold's composition, correlating with the increase in calcium concentration leaching out from the scaffolds. The leaching of calcium ions from the scaffolds directly enhances the cell's proliferation rate due to the fact that calcium ions are known biomolecules that are essential in determining early cell behavior [30]. However, the proliferation rate of cells cultured in the extracts of scaffold 4 with the highest content of nano cockle shell powder showed a drop in the number of viable cells compared to scaffolds 1, 2, and 3. This phenomenon is likely due to the changes of pH values of the culture medium with the extracts of scaffold 4 containing higher content of calcium rendering a highly basic condition for cell growth. The extracts of scaffold 3, however, proved to be significantly favorable in facilitating higher cell proliferation rate.

### 3.10. Live/Dead Cell Staining

**Figure 8:**
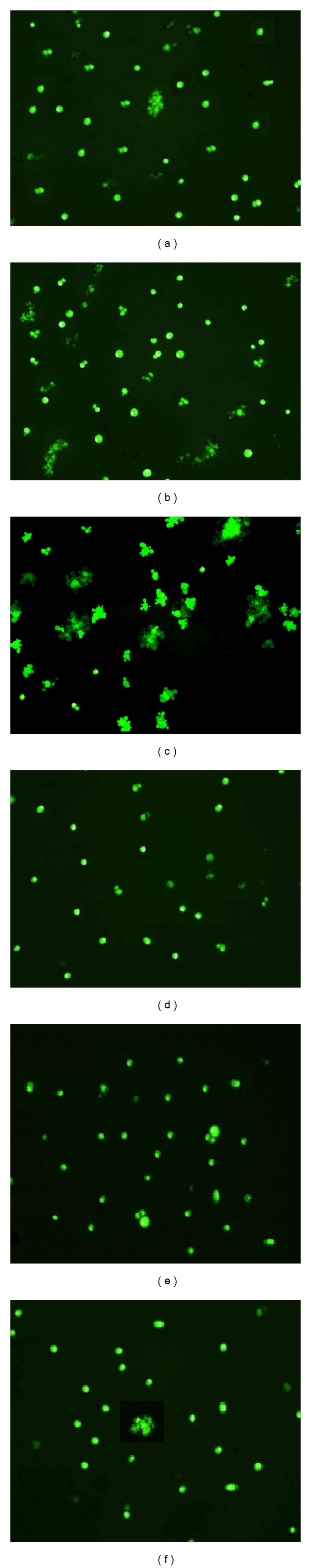
Fluorescence stained cells grown in the medium containing leachable of scaffold 1 (a), scaffold 2 (b), scaffold 3 (c), scaffold 4 (d), scaffold 5 (e), and normal culture medium (f) taken at ×10 magnification.

**Figure 9:**
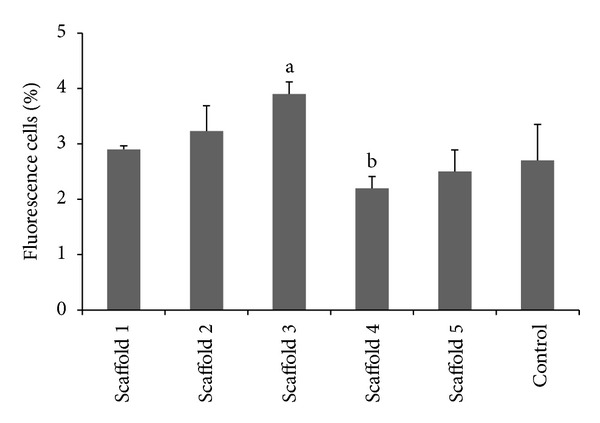
Percentage of fluorescence cells observed in different scaffold leachable as well as control medium. ^a^Significantly higher than the percentage of cells grown in leachable of all scaffolds and control at *P* < 0.05, ^b^Significantly lower than the percentage of cells grown in scaffolds 1, 2, and 3 at *P* < 0.05.

The live/dead cell staining procedure was carried out to supplement the results from the MTT assay in which the identifications of viable cells are observed to produce a green fluorescence when the stains are incorporated with the cell's DNA (Figure 8). Significantly higher percentage of cells in the culture of medium containing extracts of scaffold 3 were observed while a significant decrease in the percentage of cells was noted in the culture with extracts of scaffold 4 (Figure 9). Cells cultured in the scaffold extracts showed 100% viability during the 5 days culture period. Clustering effects of the cells correlating to the higher number of cells in medium with extracts of scaffolds 2 and 3 indicates the adhesive behavior of the MG63 cells.

## 4. Conclusion

In the present study, we have developed and characterized porous three-dimensional scaffolds using a novel combination of alginate and calcium carbonate in the form of nano cockle shell powder. Findings from this study justified the need to develop and evaluate biocomposite based scaffolds in varying composition in order to determine the most suitable composition that will ensure the success of the developed scaffold in the actual biological system. Changing the amount of polymer used in the composition mixture is necessary in determining the porous structure of the scaffolds which will ultimately determine its subsequent characteristics. From the findings of this study scaffold 3 consisting of 40% alginate and 60% nano cockle shell powder showed favorable properties in terms of its morphology, physiochemical evaluation, and mechanical strength, additionally significantly increasing cell proliferations. Cell attachment analysis and* in vivo* evaluations are being carried out in our laboratory to further justify the use of these materials as potential bone grafting substitutes.

## Figures and Tables

**Table 1:** Pore diameter range and average pore size (*μ*m) of each scaffold observed through SEM analysis.

Samples	Composition (%) Alg : nCP	Pore diameter range (*μ*m)	Average pore size (*μ*m)	Description
Scaffold 1	80 : 20	72–336	187.6 ± 63.4	Samples showed the presence of typical egg box structure of alginate with numerous rounded and well interconnected pores.
Scaffold 2	60 : 40	56–333	167.4 ± 48.1	
Scaffold 3	40 : 60	50–231	143.3 ± 36.6	

Scaffold 4	20 : 80	136–645	202.7 ± 36.9	Sample appeared to display elongated pores with overlapping sheet like structure with wide spacing between sheets. Very few rounded pores were observed.

Scaffold 5	100 : 0	76–385	198.1 ± 62.1	Sample appeared to display a lamellar sheet like structure with very few rounded pores.

Alg: alginate; nCP: nano cockle shell powder.
